# Metabolic Reprogramming in Response to Freund’s Adjuvants: Insights from Serum Metabolomics

**DOI:** 10.3390/microorganisms13030492

**Published:** 2025-02-22

**Authors:** Kiruthiga Mone, Eloy Jose Torres Garcia, Fatema Abdullatif, Mahima T. Rasquinha, Meghna Sur, Mostafa Hanafy, Denise K. Zinniel, Shraddha Singh, Raymond Thomas, Raul G. Barletta, Teklab Gebregiworgis, Jay Reddy

**Affiliations:** 1 School of Veterinary Medicine and Biomedical Sciences, University of Nebraska-Lincoln, Lincoln, NE 68583, USA; kmone2@huskers.unl.edu (K.M.); mahima.rasquinha@mssm.edu (M.T.R.); msur2@huskers.unl.edu (M.S.); mhanafy2@unl.edu (M.H.); dzinniel2@unl.edu (D.K.Z.); ssingh22@huskers.unl.edu (S.S.); rbarletta@unl.edu (R.G.B.); 2 Department of Biochemistry, Schulich School of Medicine and Dentistry, University of Western Ontario, London, ON N6A 5C1, Canada; etorresg@uwo.ca (E.J.T.G.); fabdull7@uwo.ca (F.A.); 3 Department of Immunology and Immunotherapy, Icahn School of Medicine at Mount Sinai, New York, NY 10029, USA; 4 Department of Microbiology and Immunology, Faculty of Veterinary Medicine, Cairo University, Giza 12211, Egypt; 5 Biotron Experimental Climate Change Research Centre, Department of Biology, Faculty of Science, University of Western Ontario, London, ON N6A 5B7, Canada; rthoma2@uwo.ca; 6 Department of Oncology, Schulich School of Medicine and Dentistry, University of Western Ontario, London, ON N6A 5W9, Canada

**Keywords:** adjuvants, metabolites, immunometabolism, biomarkers, immune response, immune regulation

## Abstract

Freund’s adjuvants have been used in vaccine and autoimmune settings, and their effects can be overlapping or unique to each. While both incomplete Freund’s adjuvants (IFA) and complete Freund’s adjuvants (CFA) influence antibody and T cell responses, the robust T helper 1 cytokines induced by the mycobacterial components make CFA the powerful immunostimulating adjuvant. In these studies, the adjuvant effects are investigated in a select population of cells, and the changes, if any, with the metabolic alterations in the systemic compartment are unclear. We investigated whether the effects of IFA and CFA can be influenced by the metabolic shifts in mice immunized with saline, IFA, or CFA using *Mycobacterium tuberculosis* var. *bovis* Bacillus Calmette–Guérin (BCG) as a positive control. After seven days of immunization, we analyzed the serum metabolite profiles using liquid chromatography coupled with high-resolution mass spectrometry and multivariate statistical analysis to identify metabolic features between the groups. The data revealed that, in the scores space, the CFA and BCG groups were more closely aligned compared to the saline group, while the IFA group displayed an intermediate profile. Furthermore, comparisons between the CFA and BCG groups showed more significant perturbations in lipid and amino acid metabolism, particularly involving glycerophospholipids, cysteine, and aromatic amino acids. In contrast, comparisons between the BCG and IFA groups indicated a more pronounced disruption in central energy metabolism pathways, such as the citric acid cycle and pyruvate metabolism. Together, the data suggest that the serum metabolite profiles in response to IFA and CFA might play a role in modulating the immune responses.

## 1. Introduction

Adjuvants are traditionally used to enhance immune responses to vaccines. The water-in-oil adjuvants containing mannide monooleate, as in the case of incomplete Freund’s adjuvant (IFA), have been used in experimental and clinical research [1]. For instance, IFA has been shown to enhance the immunogenicity of vaccines, including those targeting melanoma and cervical cancer vaccine [2,3]. Although IFA immunizations result in strong antibody responses, their inability to induce a strong T helper (Th)1 response was a drawback. This limitation can be addressed with the incorporation of *Mycobacterium tuberculosis* (*M. tb*) extract in the complete Freund’s adjuvant (CFA) formulation [4]. Nonetheless, cutaneous reactions and organ toxicity, especially associated with CFA, restrict their use in translational settings but are valuable to determine the immune responses experimentally, including those in autoimmune studies [5,6,7,8,9].

The use of IFA and CFA has been investigated in various autoimmune disease models such as experimental autoimmune encephalomyelitis, type I diabetes (TID), and arthritis, among others [4]. Essentially, both IFA and CFA contain the same oily base, mannide monooleate, with an extract derived from the *M. tb* H37Ra strain as an additional ingredient in the CFA [10]. In autoimmune settings, it is widely accepted that the administration of self-antigens emulsified in CFA induces disease resulting from the induction of Th1 and Th17 responses [7,11]. However, similar administrations of IFA do not induce disease, an effect initially attributed to tolerance, but later identified to be due to the induction of Th2 responses [1].

While these outcomes are generally consistent with most autoimmune disease models, a few unexpected observations have been reported. For example, administration of IFA or CFA emulsions alone without antigens can lead to the development of arthritis in rats [4]. Conversely, administration of CFA emulsion into non-obese diabetic mice can prevent the progression of TID [12]. Similarly, the anti-tuberculosis vaccine *Mycobacterium tuberculosis* var. *bovis* Bacillus Calmette–Guérin (BCG) can also prevent TID development [13]. These observations suggest that the underlying mechanisms of the effects of IFA and CFA might be complex and appear not to be due to the presence or absence of *M. tb* components alone.

Mechanistically, the adjuvant effects are attributed to the slow release of antigens and cytokine production in innate immune cells accompanied by the non-specific proliferation of antigen-reactive lymphocytes, which culminate in the induction of effective adaptive immune responses [4]. However, most studies on adjuvants are largely investigated in a select population of cells, and whether adjuvants could induce systemic metabolic responses remains unclear. Metabolomics is the comprehensive analysis of metabolites within a biological system, capturing changes in response to disease, environmental factors, medications, or toxins. As downstream products of all other “omics” (genomics, transcriptomics, and proteomics), metabolites could be directly linked to the phenotype in question [14]. This makes metabolomics a powerful tool for studying the systemic effects of adjuvants beyond traditional immunological endpoints as it identifies metabolic alterations and key pathways involved in immune responses [15,16,17].

Immune cells predominantly circulate through blood and lymphatic systems, actively exchanging metabolites with the surrounding environment [18,19,20]. Serum metabolomics, therefore, can provide a snapshot of systemic metabolic changes, reflecting immune cell activity and metabolic crosstalk in response to immune-stimulating factors such as adjuvants. Recent evidence suggests that small molecules could target metabolic pathways such as mevalonate and the mammalian target of rapamycin (mTOR) that can regulate immune responses [21,22,23,24]. For example, metabolites, such as kynurenine, a tryptophan metabolite produced by the enzyme indoleamine 2,3-dioxygenase 1 (IDO1), could stimulate antigen-presenting cells (APCs) [25]. However, investigations into the serum or plasma metabolite changes induced by adjuvants have been rarely reported. In this study, we provide a comprehensive analysis of serum metabolites in different adjuvants. We used liquid chromatography coupled with high-resolution mass spectrometry (LC-MS) and multivariate statistical analysis to determine the metabolites in the sera obtained from A/J mice immunized with or without IFA and CFA or BCG independent of any antigenic stimulations. The analysis revealed that the metabolites of CFA and BCG aligned together, showing pronounced alterations in the lipid and amino acid metabolisms, whereas those of the IFA group had mixed profiles, suggesting that the select metabolites could be used as metabolomic biomarkers.

## 2. Materials and Methods

### 2.1. Mice

Six-week-old female A/J mice (H-2^a^) were procured from Jackson Laboratory (Bar Harbor, ME, USA) and maintained according to the Institutional Animal Care and Use Committee’s guidelines of the University of Nebraska-Lincoln (protocol #: 2321), Lincoln, NE, USA. BCG immunization studies were performed based on biosafety level 2 guidelines. Animals were euthanized using a carbon dioxide chamber as recommended by the Panel on Euthanasia of the American Veterinary Medical Association.

### 2.2. BCG Propagation and Enumeration

A loopful of *Mycobacterium tuberculosis* var. *bovis* BCG str. Pasteur 1173P2 frozen stock was streaked on Middlebrook 7H9 (Becton Dickson, Sparks, MD, USA) plates supplemented with 10% Middlebrook Oleic Acid-Albumin-Dextrose-Catalase (OADC) (Becton Dickson, Sparks, MD, USA) and 1.5% agar. The plates were incubated at 37 °C. After the plates grew visible colonies, a single BCG colony was transferred to 10 mL of Middlebrook 7H9 broth supplemented with 10% OADC. The broth was incubated at 37 °C in a shaking incubator for 1 week and then scaled up to 100 mL. After the BCG broth reached an optical density (OD)_600_ of 0.8, the media was centrifuged at 3000× *g* for 30 min. The bacterial pellet was resuspended in 10 mL of freezing media (0.9% sodium chloride and 10% glycerol) then aliquoted into 2 mL tubes and frozen at −80 °C for storage. Two weeks before the inoculation of the mice, one aliquot was thawed, diluted, and plated on 7H9 media supplemented with 10% OADC and 1.5% agar to obtain final colony-forming units (CFU) after freezing. For in vivo experiments, 5 × 10^6^ CFU/animal of BCG was administered intravenously in 200 µL.

### 2.3. Immunization

For immunizations, we used IFA, CFA (Sigma-Aldrich, St. Louis, MO, USA) supplemented with the heat-killed extract of *M. tb* H37Ra (Difco Laboratories, Detroit, MI, USA) in a 5 mg/mL concentration, and BCG. Groups of animals were administered subcutaneously with 200 µL of saline, IFA, and CFA emulsions as a single dose on day 0 into the shoulder and hip regions, whereas BCG (5 × 10^6^ CFU/animal) was administered intravenously via the tail vein. Seven days later, animals were euthanized, blood was collected by cardiac puncture, and serum was harvested.

### 2.4. Metabolite Extraction for Serum Sample

Three technical replicates were prepared from each biological sample. Polar and semi-polar metabolites were extracted from the serum matrix using cold methanol: water (80:20). The serum samples were centrifuged at 12,000× *g* for 5 min; 100 µL of the supernatant was transferred to a 1.5 mL microfuge tube containing 400 µL of pre-chilled (−20 °C) methanol (80:20); followed by 15 min centrifugation at 12,000× *g*, 50 µL of the supernatant was transferred to an LC vial for LC-MS analysis [26,27].

### 2.5. LC-MS Data Acquisition and Pre-Processing

All samples were analyzed using the Thermo Q-Exactive Orbitrap® mass spectrometer (Thermo Scientific, Waltham, MA, USA) coupled to an Agilent 1290 high-performance liquid chromatography (HPLC) system (Agilent Technologies, CA, USA). The instrumental conditions for heated electrospray ionization were a capillary voltage of 3.9 kV; capillary temperature, 400 °C; sheath gas, 17 arbitrary units; auxiliary gas, 8 units; probe heater temperature, 450 °C, and S-Lens RF level, 45%. Hydrophilic interaction liquid chromatography (HILIC) analysis was performed in both positive and negative ionization modes [28]. In both cases, 2 μL of the sample was injected onto an Agilent HILIC-Z, peek-line column (2.1 × 100 mm, 2.7 μm; Agilent, Santa Clara, CA, USA) kept at 35 °C. Metabolites were eluted with mobile phases of 20 mM ammonium formate in water (A) and 20 mM ammonium formate in 90% acetonitrile (B) operating with the following gradient: 0 min, 100% B; 0.5 min, 100% B; 5.3 min, 80% B; 9.5 min, 30% B; 13.5 min, 30% B, 14.5 min 100% B, and 16.5 min, 100% B. The HILIC acquisition mode was a top 5 data-dependent acquisition experiment composed of a full MS scan in the mass range of *m*/*z* 70–1000 at 35,000 resolutions, automatic gain control (AGC) target of 5 × 10^5^, maximum injection time (maxIT) of 128 ms, followed by 5 MS/MS scans at 17,500 resolution, isolation window of *m*/*z* 1.2, normalized collision energy of 35, AGC target of 3 × 10^6^, maxIT of 64 ms, intensity threshold of 1.3 × 10^5^, and dynamic exclusion of 10 s [28]. Thermo raw data output files were converted to mzML format using Proteowizard 3.0 [29] with peak-picking filtering. All the spectral features were detected and aligned using the MZmine 4.3.0 package batch processing [30].

### 2.6. Metabolomics Data Processing and Statistical Analysis

The LC-MS peak lists were processed and analyzed using MetaboAnalyst 6.0 (http://www.metaboanalyst.ca) accessed on 10 November 2024 [31,32]. Before the multivariate analysis, the features with greater than 50% missing values across the dataset samples were excluded from the data. The remaining missing values were imputed using a value equivalent to 20% of the lowest detected peak area for that specific metabolite feature across all samples in the dataset. Then, the peak list was filtered by removing LC-MS peaks that were near-constant throughout the experiment condition, employing a 40% interquartile range [33]. The samples were then normalized by total ion count and log-transformed, and autoscaling were applied [14]. We used principal component analysis (PCA) for unsupervised multivariate analysis to investigate cluster separation that is indicative of the metabophenotype. Partial least squares discriminant analysis (PLS-DA) was applied to identify important variables with discriminative power for projection. The variable importance in projection (VIP) was used to select the features that discriminate the class. Pathway analysis was performed by MetaboAnalyst 6.0 using the Kyoto Encyclopedia of Genes and Genomes (KEGG) pathway of *Mus musculus* [34]. The PCA and PLS-DA scores were used to generate a metabolomics tree diagram using the PCAtoTree tool 1.0 [35].

### 2.7. Metabolite Annotation

The LC-MS peaks identified by the VIP scores plots were then annotated following the confident level system proposed by Reisdorph et al., 2019 [36]. According to the confident level system, this study was performed at level 3 (medium: putatively annotated compound conferring chemical properties that led metabolite class based on the comparison of parent *m*/*z* and retention time or MS1 analysis), level 2 (high: identified metabolites using the MS/MS libraries on the merit of the fragmentation pattern of ions in MS2), and level 1 (highest, identified matching to authentic standards) [27,36]. When standards were not available for level 1 assignment, the online MassBank of North America (MoNA, http://mona.fiehnlab.ucdavis.edu/)(accessed on 28 December 2024) and MassBank Europe (https://massbank.eu/MassBank/) (accessed on 28 December 2024) databases were used to identify metabolite features, by matching detected *m/z* with those from the database (5 parts per million [ppm] error tolerance) using SIRIUS 4 (https://bio.informatik.uni-jena.de/sirius/) (accessed on 28 December 2024) [37]. Annotated metabolites and their corresponding confidence levels are listed in Supplementary Table S1.

## 3. Results and Discussion

### 3.1. Serum Adjuvants Have Distinct Metabolic Phenotypes

In this report, we describe the comparative serum metabolomic profiles in response to adjuvants using the untargeted LC-MS approach that enabled us to conduct unbiased analysis of the comparative metabolite changes induced by IFA, CFA, and BCG. Essentially, the metabolomic analysis is performed using targeted or untargeted approaches. While the targeted metabolomics is limited to the detection of select compounds derived by perceived hypothesized metabolic changes [38,39], the untargeted approach permits discovery research in an unbiased manner [14]. In this study, we conducted a comprehensive evaluation of serum metabolites in response to IFA and CFA that are routinely used in experimental and clinical research.

Fundamentally, IFA is a water-in-oil emulsion containing the surfactant mannide monooleate in mineral oil [40], whereas CFA contains heat-killed mycobacterial extract, in particular *M. tb* H37Ra, as an additional ingredient [10]. Because of the mycobacterial content, the adjuvanticity of CFA is unparalleled in inducing robust immune responses that include antibody and cell-mediated immune (CMI) responses [41]. Nonetheless, the translational use of CFA is limited to none owing to toxicity and severe local reactions [5,6,9]. Conversely, although relatively safe, the use of IFA is also limited due to its inability to induce strong CMI responses [1]. Regardless of these limitations, the mechanistic understanding of IFA and CFA at a systemic level is largely unknown, and most studies involve the determination of their effects at a cellular level that may include innate and adaptive immune cells [4,42,43,44,45]. Since the immune cell activation processes can influence immune cell metabolism, the serum serves as a microenvironment for circulating immune cells; we sought to investigate whether serum metabolites are induced distinctly by different adjuvants [46,47]. In this setting, we included BCG as a positive control. Of note, BCG is a live attenuated *M. bovis* as opposed to killed *M. tb* extract in CFA [10,48]. Thus, the use of IFA, CFA, and BCG facilitate the identification of distinct metabolites, if any, in IFA in relation to CFA and BCG.

**Figure 1:**
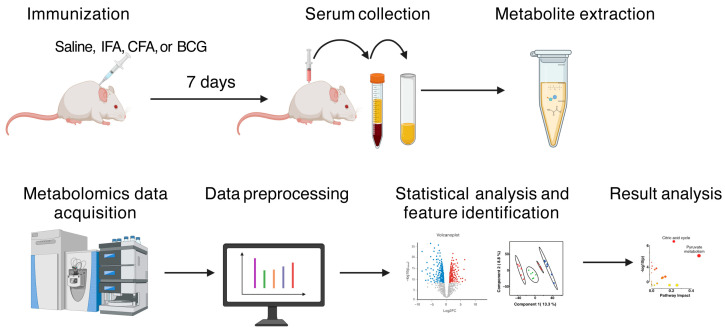
Metabolomics workflow to investigate metabolic profiles in response to immunizations by HILIC high-resolution mass spectrometry. Figure created with BioRender.com (accessed on 24 January 2024).

We immunized groups of A/J mice with IFA, CFA, and BCG, six animals per group, except for IFA, which consists of four animals. We used saline as a negative control. After seven days, sera were collected and subjected to untargeted LC-MS analysis. The metabolomics data were acquired using a Thermo Q-Exactive Orbitrap^®^ mass spectrometer coupled with Agilent 1290 HPLC (Figure 1). Instrument performance during the experiment was validated using phenylalanine (for positive ionization) and succinic acid (for negative ionization). The mass tolerance for phenylalanine and succinic acid remained stable at 5 ppm across multiple samples (Supplementary Figures S1 and S2).

**Figure 2:**
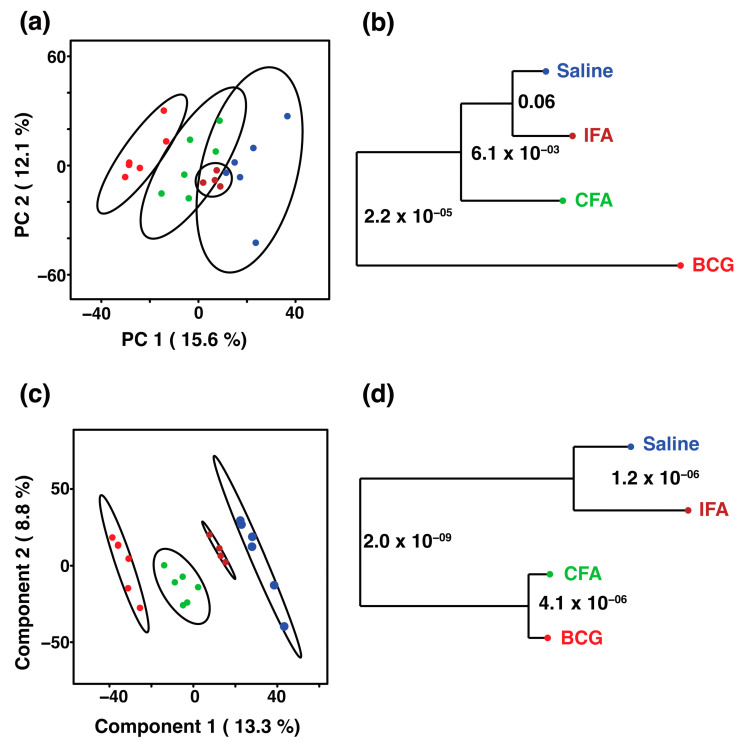
Multivariate analysis of serum metabolites in response to immunizations. (**a**) The 2D PCA and (**c**) 2D PLS-DA score plots generated from the LC-MS data of sera collected from four treatment groups: saline (blue 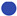), IFA (brown 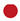), CFA (green 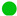), and BCG (red 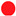). The ellipsis represents the 95% confidence limit from a normal distribution for each cluster. The predictive ability of the PLS-DA data were measured by cross-validation, demonstrating high predictive performance with an explained variance of R^2^ = 0.99 and a predictive variance of Q^2^ = 0.98. (**b**,**d**) Metabolomics tree diagram generated from the scores plot of the PCA and PLS-DA, respectively. The numbers indicate the *p*-value for each node separation. The coloring of each group in the tree diagram is similar to the scores plot.

To study the pattern of serum metabolic response in different treatment groups, we used an unsupervised principal component analysis. The PCA scores plot of the LC-MS data illustrate the metabolic changes in the sera collected from different groups (Figure 2a). As indicated on the PCA scores plot, the metabolites of the BCG group form a distinct cluster in the PCA scores space, indicating a unique metabolic phenotype compared to the other groups. The cluster of the CFA group lies between the BCG and IFA groups, suggesting shared features of both groups. Although the saline group forms a separate cluster, the samples appeared to overlap with the IFA group (Figure 2a). To further understand and validate the differences in the clusters observed in the PCA scores plot (Figure 2a), we generated a metabolomics tree diagram using the PCAtoTree program [35]. The program uses the Mahalanobis distance metric (*p*-values) to assess the statistical significance of the cluster classification. The PCAtoTree tree diagram revealed that BCG has the most discernable effect, forming a separate branch in the tree diagram with a *p*-value of 2.2 × 10^−5^ (Figure 2b). The CFA group has moderate effects on altering the serum metabolic profiles related more closely to the BCG group but significantly distinct from the IFA and saline groups, with a *p*-value of 6.1 × 10^−3^. The IFA and saline groups were found in the same branch with a marginal difference (*p*-value = 6 × 10^−2^) (Figure 2b). To further verify our observations, we performed a supervised PLS-DA analysis of the LC-MS data between treatment groups (Figure 2c). The PLS-DA scores plot shows a clear separation between groups, since the supervised method is optimized for group separation, requiring validation of the predictive ability of the model. To assess the predictive ability of the PLS-DA model in distinguishing between groups, we conducted a cross-validation. The high R^2^ (0.99) and Q^2^ (0.98) values indicate a strong predictive performance. Notably, the PLS-DA scores plot emphasizes that the serum metabolite alterations are due to adjuvant effects. In the PLS-DA scores space, the IFA-immunized group lies closer to the saline group, followed by the CFA and BCG groups. The BCG group exhibited the most distinct metabolite profile compared to the saline group (Figure 2c). To assess the statistical significance of the observed group separation, we used the PCAtoTree program to generate a metabolite tree diagram with a *p*-value using the Mahalanobis distance metric (Figure 2d). The tree diagram clusters the CFA and BCG groups in one branch, demonstrating their metabolic profile to be distinct from those of the IFA and saline groups (*p*-value = 2 × 10^−9^). Further branching between the CFA and BCG groups revealed metabolomics profiles to be distinct from each other, as indicated in the tree diagram in which both groups branched out independently with a *p*-value of 4.1 × 10^−6^. Similarly, the saline and IFA groups branched out independently with a *p*-value of 1.2 × 10^−6^ (Figure 2d). Overall, both the unsupervised and supervised models revealed that the BCG and CFA groups aligned closer to each other, as much as IFA and saline, suggesting that their metabolite compositions could be different.

### 3.2. BCG Reprograms Serum Metabolites Associated with Energy Metabolism

**Figure 3:**
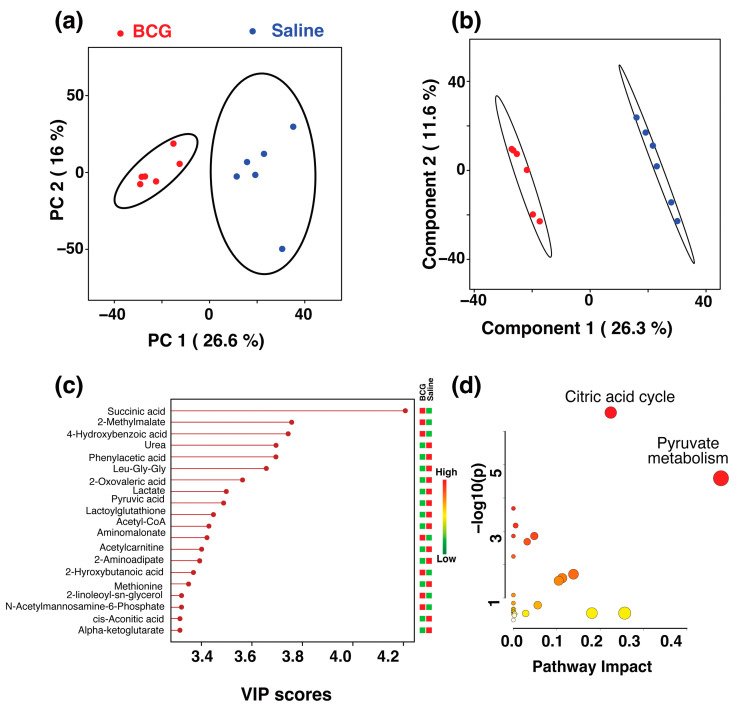
Serum metabolic alterations induced by BCG. (**a**) The 2D PCA and (**b**) 2D PLS-DA score plots generated from the LC-MS data of sera collected from saline (blue 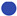) and BCG (red 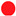) groups. The ellipsis represents the 95% confidence limit from a normal distribution for each cluster. The predictive ability of the PLS-DA data were measured by cross-validation, demonstrating high predictive performance with an explained variance of R^2^ = 0.99 and a predictive variance of Q^2^ = 0.9. (**c**) VIP scores for metabolites that best differentiate the BCG from the saline group. Higher VIP scores indicate metabolites with greater discriminative power in the model. The top 20 metabolites are listed along the *y*-axis, with their respective VIP scores on the *x*-axis. The colored squares to the right indicate the relative levels of each metabolite in the BCG and saline groups, with a gradient color bar showing low (green) to high (red) intensity. (**d**) Pathway analysis based on metabolites contributing to the separation between BCG and saline groups, as identified in (**c**). The two major impacted pathways, namely, the citric acid cycle and pyruvate metabolism, as analyzed by using *Mus musculus* KEGG analysis, are shown.

We sought to identify the metabolites, and the analyses revealed distinct phenotypes associated with each adjuvant. First, we compared the metabolites of the BCG group with those of saline recipients. Consistent with the group analysis (Figure 2), the LC-MS features of the BCG and saline groups clustered into two different clusters in a PCA scores plot where the primary separation is on the first principal component (Figure 3a). To identify the metabolites contributing to the class separation, we built a PLS-DA model (Figure 3b). Among the top 20 metabolites that contributed to the class separation, we noted an elevation pattern of seven metabolites in the BCG group compared to the saline group. These include succinic acid, 2-methylmalate, 4-hydroxybenzoic acid, aminomalonate, 2-hydroxybutanoic acid, methionine, and N-acetylmannosamine-6-phosophate (Figure 3c). Further, pathway analysis of these altered metabolites revealed that the major pathways impacted in the BCG groups were the citric acid cycle and pyruvate metabolism (Figure 3d). Reports indicate the detection of other metabolites, such as lactate, and glutamate in BCG recipients, especially at a cellular level [49], which were not apparent in our studies. These variations could be due to BCG strains used in different studies [50,51,52].

### 3.3. Minimal Metabolite Perturbation Between BCG and CFA

**Figure 4:**
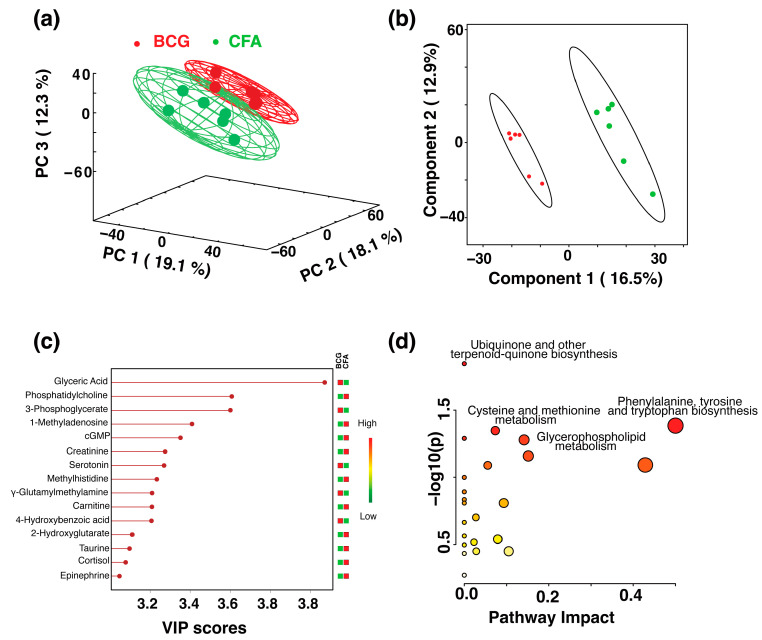
Comparison of serum metabolomic profiles between the BCG and CFA groups. (**a**) The 3D PCA score plot showing the separation of metabolic profiles between the BCG (red 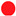) and CFA (green 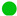) groups. The percentage of variance explained by each principal component is indicated on the respective axes (PC1: 19.1%, PC2: 18.1%, PC3: 12.3%). The ellipsoids represent a 95% confidence limit from a normal distribution for each cluster. (**b**) The 2D PLS-DA score plot showing a clear separation between the BCG and CFA metabolic profiles along Component 1 (16.5%) and Component 2 (12.9%) with cross-validation values of R^2^ = 1 and Q^2^ = 0.54. (**c**) VIP scores for metabolites that best differentiate the BCG from the CFA group. Higher VIP scores indicate metabolites with greater discriminative power in the model. The top 15 metabolites are listed along the *y*-axis, with their respective VIP scores on the *x*-axis. The colored squares to the right indicate the relative levels of each metabolite in the BCG and CFA groups, with a gradient color bar showing low (green) to high (red) intensity. (**d**) Pathway analysis based on metabolites contributing to the separation between BCG and CFA groups, as identified in (**c**). The major impacted pathways, namely, the ubiquinone and other terpenoid-quinone biosynthesis, cysteine and methionine metabolism, phenylalanine, tyrosine, and tryptophan biosynthesis, and glycerophospholipid metabolism as analyzed by using *Mus musculus* KEGG analysis are shown.

We then compared the metabolites between BCG and CFA groups. We generated a 3D PCA plot that shows a separation between the two groups (Figure 4a). Then, we used the PLS-DA model (Figure 4b) and its VIP score plot to reveal the metabolites that contribute to the cluster difference between the BCG and CFA groups (Figure 4c). Out of the 15 metabolites that contribute to the class separation, we noted eight of them were higher in the CFA group when compared with the BCG group (Figure 4c). They were noted in the order of their VIP score phosphatidylcholine, 1-methyladenosine, cGMP, creatinine, methyhistidine, carnitine, 2-hydroxyglutarate, and taurine, including cortisol and epinephrine. By relating these altered metabolites to metabolic pathways, we noted ubiquinone and other terpenoid-quinone biosynthesis, cysteine and methionine metabolism, phenylalanine, tyrosine, and tryptophan biosynthesis, and glycerophospholipid metabolism (Figure 4d). Of note, both BCG and CFA possess similar mycobacterial components, but their nature is different (live vs. killed), which may be the reason for variations noted between the two groups [10,48]. Alternatively, the surfactant and the mineral oil base in the CFA might be a contributing factor for elevated metabolites noted in this group.

### 3.4. Multivariate Analysis of BCG and IFA Serum Metabolites

**Figure 5:**
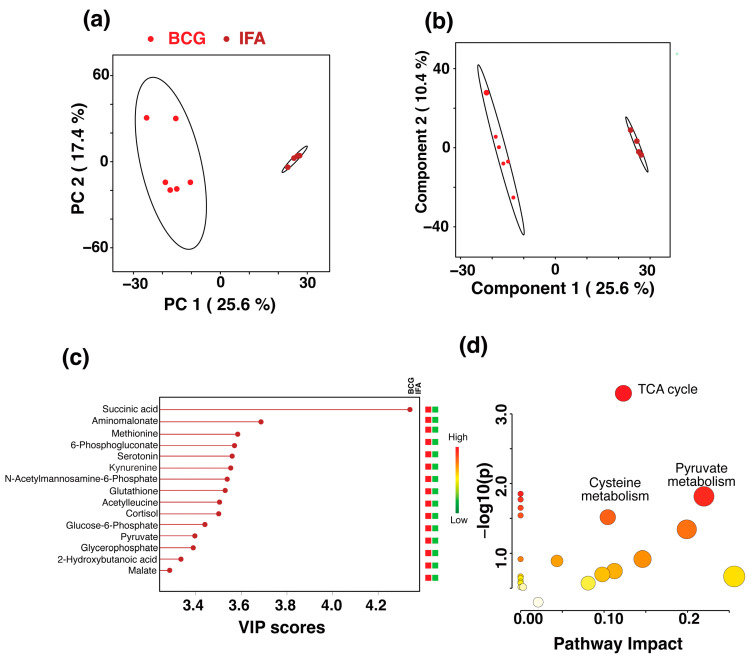
Comparison of serum metabolomic profiles between the BCG and IFA groups. (**a**) The 2D PCA score plot showing the separation of metabolic profiles between the BCG (red 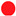) and IFA (brown 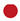) groups. The ellipses represent a 95% confidence limit from a normal distribution for each cluster. (**b**) The 2D PLS-DA score plot showing a clear separation between the BCG and IFA metabolic profiles along the two components with cross-validation values of R^2^ = 0.99 and Q^2^ = 0.98 (**c**) VIP scores for metabolites that best differentiate the BCG from the IFA group. Higher VIP scores indicate metabolites with greater discriminative power in the model. The top 15 metabolites are listed along the *y*-axis, with their respective VIP scores on the *x*-axis. The colored squares to the right indicate the relative levels of each metabolite in the BCG and IFA groups, with a gradient color bar showing low (green) to high (red) intensity. (**d**) Pathway analysis based on metabolites contributing to the separation between the BCG and IFA groups, as identified in (**c**) The two major impacted pathways, namely, the citric acid cycle, cysteine metabolism, and pyruvate metabolism, as analyzed by using *Mus musculus* KEGG analysis, are shown.

To analyze the metabolites that could be potentially altered by the surfactant/mineral oil, we compared the metabolites between the BCG and IFA groups. The data revealed distinct clusters as analyzed by PCA (Figure 5a) and PLS-DA scores plot (Figure 5b), which has R^2^ = 0.99 and Q^2^ = 0.98 values. The top 13 metabolites accounted for the PLS-DA cluster difference that were all elevated in the BCG group were in the order of their VIP scores: succinic acid, aminomalonate, methionine, 6-phosphogluconate, kynurenine, N-acetylmannoseamine-6-phosphate, glutathione, acetylleucine, glucose-6-phosphate, pyruvate, glycerophosphate, 2-hydroxybutanoic acid, and malate, in addition to serotonin and cortisol (Figure 5c). The major pathways impacted by these metabolites were the Tricarboxylic acid (TCA) cycle, cysteine metabolism, and pyruvate metabolism (Figure 5d). Similar analysis between CFA and IFA groups did not reveal significant variations (Supplementary Figure S3), suggesting that subtle variations could be due to the *M. tb* extract in the CFA group, and surfactant/mineral oil appear not to greatly influence serum metabolic profiles. This proposition is supported by the observation that the metabolic clusters were comparable between IFA and saline groups (Figure 2).

Overall, our data revealed that the metabolites induced by BCG were distinct from the saline and IFA groups, whereas those of CFA and BCG were similar. The notable metabolites upregulated in the BCG group compared to the saline or IFA group were succinic acid and methionine (Table 1). While succinic acid, an intermediary of the TCA cycle, has a role in metabolic reprogramming and epigenetic changes and modulates inflammatory responses [53,54,55], methionine is involved in lipid metabolism and cell death involving lipid peroxidation [56,57,58]. Likewise, the elevated levels of 4-hydroxybenzoic acid in the BCG group could modulate nucleotide-binding domain, leucine-rich repeat, and pyrin domain-containing protein-3 (NLRP3) inflammasome activation and oxidative stress [59,60]. Among the metabolites upregulated in the BCG group in relation to the IFA group, the roles of kynurenine and glutathione are well documented (Table 1). For example, kynurenine, the metabolite of tryptophan metabolism involving IDO1, could mediate inflammatory responses, oxidative stress, and immune suppression in addition to causing endothelial dysfunction [61,62,63]. Similarly, glutathione, as an antioxidant and signaling molecule, could modulate natural killer (NK) cell and T cell responses [64,65,66,67,68,69,70]. Other metabolites upregulated in this group, such as glycerophosphate, glucose-6-phosphate, pyruvate, and malate, are involved in energy metabolism [71,72,73,74]. However, none of the metabolites related to the central energy metabolism remain unaffected in the CFA group compared to the BCG group (Table 1). Nonetheless, the metabolites upregulated in the CFA group, such as phosphatidylcholine, creatinine, carnitine, and 2-hydroxyglutarate, have been reported to mediate anti-inflammatory functionalities [75,76,77,78,79,80,81,82,83,84,85,86,87,88,89,90,91,92,93]. In addition, 1-methyladenosine and taurine, including 2-hydroxyglutarate, have been identified as oncometabolites [91,94,95,96,97,98,99,100] (Table 1). Taken together, our data provide evidence that the metabolites induced by adjuvants can be captured systemically in serum. Since we used an untargeted LC-MS approach to determine the metabolite compositions in an antigen-free adjuvant systems, the metabolites noted in our studies could influence the immune responses to antigens emulsified in Freund’s adjuvants that may involve a combination of different functions described above.

## Figures and Tables

**Table 1:** Metabolic alterations in response to adjuvants.

Metabolite	Major Role	Ref.
Upregulated metabolites in the BCG group as compared to both saline and IFA groups		
Succinic acid	Intermediate of TCA cycle, metabolic reprogramming, epigenetic regulator and modulator of inflammatory response	[53,54,55]
Aminomalonate	Amino acid synthesis	[101]
2-hydroxybutanoic acid	Biomarker of preclampsia	[102]
Methionine	Lipid metabolism and ferroptosis	[56,57,58]
N-acetylmannosamine-6-phophate	Sialic acid synthesis	[103]
Upregulated metabolites in the BCG group as compared to saline group		
4-hydroxybenzoic acid	NLRP3 inflammasome activation and oxidative stress	[59,60]
Upregulated metabolites in the BCG group as compared to IFA group		
6-phosphogluconate	Reprogramming of Treg cells	[104]
Kynurenine	Inflammation, oxidative stress, endothelial dysfunction and immune suppression	[61,62,63]
Glutathione	Antioxidant, signaling and NK cell and T cell responses	[64,65,66,67,68,69,70]
Acetylleucine	mTOR pathway	[105]
Glycerophosphate	Adenosine triphosphate synthesis	[71]
Glucose-6-phosphate	Central energy metabolism	[72]
Pyruvate	Central energy metabolism, antioxidant and suppression of T cell responses	[73]
Malate	TCA cycle intermediate	[74]
Upregulated metabolites in the CFA group as compared to BCG group		
Phosphatidylcholine	Anti-inflammatory	[75]
1-methyladenosine	Oncometabolite	[94,95]
cGMP	Innate immune signaling	[106,107,108,109,110]
Creatinine	Anti-inflammatory, immunomodulator and antioxidant	[76,77,78]
Methylhistidine	Biomarker of skeletal muscle breakdown	[111]
L-carnitine	Downregulate T cell responses and block inflammation	[79,80,81,82,83]
2-hydroxyglutarte	Oncometabolite, promotes Treg cells, regulates T cell response, anti-inflammatory and blocks NF-AT activation	[84,85,86,87,88,89,90,91,92,93]
Taurine	Oncometabolite, pro- and anti-inflammatory	[96,97,98,99,100]

## Data Availability

The original contributions presented in this study are included in the article/supplementary material. Further inquiries can be directed to the corresponding authors.

## Supplementary Materials

The following supporting information can be downloaded at: https://www.mdpi.com/article/10.3390/microorganisms13030492/s1, Figure S1: Representative chromatogram of phenylalanine illustrating the instrument’s performance during the experiment. The chromatograms demonstrate consistent and reliable detection of phenylalanine (neutral mass: 165.07898, *m*/*z*: 166.0861 [M+H]+, formula: C_9_H_11_NO_2_) across multiple sample injections. The base peak intensity observed at the expected retention time (RT~4.92 min) highlights the stability and reproducibility of the LC-MS system operating in HILIC-positive ionization mode. Detection remained within the defined *m*/*z* tolerance of 5 ppm, ensuring accurate mass identification and high precision. The reproducible retention times and consistent peak intensities across all analyzed samples validate the robustness and reliability of the LC-MS system. These results confirm the system’s consistent performance, enabling high-quality data acquisition; Figure S2: Representative chromatogram of succinic acid illustrating the performance of the instrument during the experiment. The chromatograms demonstrate consistent and reliable detection of succinic acid (neutral mass: 118.026086, *m*/*z*: 117.01934 [M-H]⁻, formula: C_4_H_6_O_4_) across multiple injections. The base peak intensity observed at the expected retention time (RT~1.52 min) highlights the stability and reproducibility of the LC-MS system operating in HILIC-negative ionization mode. The peaks are well-aligned within the defined m/z tolerance of 5 ppm, ensuring accurate mass identification and confirming system precision; Figure S3: (a) Three-dimensional PCA plot showing the distribution of IFA and CFA samples with corresponding ellipses representing 95% confidence interval. (b) PCAtoTree diagram generated from PCA scores, illustrating the minimal distinction between IFA and CFA groups with *p*-value of (0.01). Table S1: List of metabolites annotated. This table summarizes the detection of metabolites using a HILIC-LC-MS method. Each row provides details of the metabolite’s neutral mass, precursor *m*/*z*, retention time (RT), molecular formula, adduct, and name. All metabolites were detected with an RT tolerance of 1.0 min in the LC phase (elution) and 5.0 ppm in the MS phase for putative identification and spectral matches. The metabolites were annotated using the confidence level system proposed by Reisdorph et al., 2019 [36]. According to this system, the study was conducted at three levels of confidence: Level 1, the highest confidence, where metabolites were identified by matching to authentic standards; Level 2, high confidence, where metabolites were identified using MS/MS libraries based on the fragmentation patterns of ions in MS2; and Level 3, medium confidence, where putatively annotated compounds were identified based on chemical properties, metabolite class, parent *m*/*z*, and retention time or MS1 analysis [27,36].

## Abbreviations

The following abbreviations are used in this manuscript:

AGC	Automatic gain control
APCs	Antigen-presenting cells
BCG	Bacillus Calmette–Guérin
CFA	Complete Freund’s adjuvant
CFU	Colony-forming units
CMI	Cell-mediated immune
HILIC	Hydrophilic interaction liquid chromatography
HPLC	High-performance liquid chromatography
IDO1	Indoleamine 2,3-dioxygenase 1
IFA	Incomplete Freund’s adjuvant
KEGG	Kyoto Encyclopedia of Genes and Genomes
LC-MS	Liquid chromatography coupled with high-resolution mass spectrometry
maxIT	Maximum injection time
*M. tb*	*Mycobacterium tuberculosis*
mTOR	Mammalian target of rapamycin
NK	Natural killer
NLRP3	Nucleotide-binding domain, leucine-rich repeat, and pyrin domain-containing protein-3
OADC	Oleic Acid-Albumin-Dextrose-Catalase
OD	Optical Density
PCA	Principal component analysis
PLS-DA	Partial least squares discriminant analysis
PPM	Parts per million
RT	Retention Time
TCA	Tricarboxylic acid
Th	T helper
TID	Type I diabetes
VIP	Variable importance in projection
