# Supplementary material for: Metabolic Reprogramming in Response to Freund’s Adjuvants: Insights from Serum Metabolomics
Source: Microorganisms. 2025 Feb 22;13(3):492. doi: 10.3390/microorganisms13030492 (PMC11944801; doi:10.3390/microorganisms13030492)
Supplement: Supplementary file 1 [file microorganisms-13-00492-s001.zip › Table S1.pdf]

Supplementary Table S1. List of metabolites annotated. This table summarizes the detection of metabolites using a HILIC-LC-MS method. Each row provides details of the metabolite's neutral mass, precursor m/z, retention time (RT), molecular formula, adduct, and name. All metabolites were detected with an RT tolerance of 1.0 minute in the LC phase (elution) and 5.0 ppm in the MS phase for putative identification and spectral matches. The metabolites were annotated using the confidence level system proposed by Reisdorph et al. 2019, (36). According to this system, the study was conducted at three levels of confidence: Level 1, the highest confidence, where metabolites were identified by matching to authentic standards; Level 2, high confidence, where metabolites were identified using MS/MS libraries based on the fragmentation patterns of ions in MS2 (27,36); and Level 3, medium confidence, where putatively annotated compounds were identified based on chemical properties, metabolite class, parent m/z, and retention time or MS1 analysis.

Molecule	Neutral mass	Precursor m/z	RT	Formula	Adduct	Confident Level
Urea	60.03236	121.2	2.2	CH4N2O	[2M+H]+	**2**
Pyruvate	88.016	87.0115	5.52	C3H3O3-	[M-H]-	**2**
Lactic acid/Lactate	90.03169	89.0239	2	C3H6O3	[M-H]-	**1**
2-Hydroxybutanoic acid	104.04734	103.1042	2.85	C4H8O3	[M-H]-	**2**
Glyceric Acid	106.02661	105.0193	4.75	C3H6O4	[M-H]-	**1**
Succinic Acid	118.02661	117.0193	1.52	C4H6O4	[M-H]-	**1**
Taurine	125.01466	124.0067	5.11	C2H7NO3S	[M-H]-	**1**
Malic Acid / Malate	134.02152	133.0142	5.9	C4H6O5	[M-H]-	**3**
Phenylacetic acid	136.05243	135.1032	0.57	C8H8O2	[M-H]-	**2**
4-Hydroxybenzoic acid	138.03169	137.0224	1.07	C7H6O3	[M-H]-	**2**
alpha-ketoglutarate	146.02152	145.014	6	C5H6O5	[M-H]-	**2**
2-Methylmalate	146.02152	145.0142	3.59	C5H6O5-2	[M-H]-	**2**
Methylhistidine	169.08513	168.0761	7.74	C7H11N3O2	[M-H]-	**1**
Glycerophosphate	172.01368	171.0059	6.49	C3H9O6P	[M-H]-	**1**
Acetylleucine	173.10519	172.0979	0.84	C8H15NO3	[M-H]-	**1**
3-Phosphoglycerate	185.99294	184.9856	1.49	C3H7O7P	[M-H]-	**1**
Glucose 6-phosphate	260.02972	259.0908	5.58	C6H13O9P	[M-H]-	**1**
6-Phosphogluconate	276.02463	275.2	7.71	C6H13O10P	[M-H]-	**3**
3',5'-Cyclic Guanosine Monophosphate (cGMP)	345.04743	344.2364	0.68	C10H12N5O7P	[M-H]-	**2**

Lactoylglutathione	379.10494	378.0976	5.7	C13H21N3O8S	[M-H]-	**1**
Creatinine	113.05891	114.0659	2.3	C4H7N3O	[M+H]+	**1**
Aminomalonic acid/Aminomalonate	119.02186	120.0291	3.74	C3H5NO4	[M+H]+	**2**
2-Hydroxyglutarate (2-HG)	148.03717	149.0235	0.6	C5H8O5	[M+H]+	**2**
Methionine	149.05105	150.0583	4.31	C5H11NO2S	[M+H]+	**1**
2-Aminoadipate	161.06881	162.0762	1.64	C6H11NO4	[M+H]+	**2**
Carnitine	161.10519	162.1125	5.24	C7H15NO3	[M+H]+	**1**
Serotonin	176.09496	177.1022	3.35	C10H12N2O	[M+H]+	**1**
Tyrosine	181.07389	182.0811	4.55	C9H11NO3	[M+H]+	**1**
Epinephrine	183.08954	184.0969	1.52	C9H13NO3	[M+H]+	**1**
Acetylcarnitine	203.11576	204.1228	0.96	C9H17NO4	[M+H]+	**3**
Gamma-Glutamylleucine	260.13722	261.1444	8.01	C11H20N2O5	[M+H]+	**3**
1-Methyladenosine	281.1124	282.1195	4.48	C11H15N5O4	[M+H]+	**1**
Methylthioadenosine (MTA)	297.08956	298.0968	4.5	C11H15N5O3S	[M+H]+	**2**
N-Acetylmannosamine 6-phosphate	301.05627	302.0641	4.38	C8H16NO9P	[M+H]+	**2**
Glutathione	307.08381	308.0911	4.45	C10H17N3O6S	[M+H]+	**2**
Cortisol	362.20932	363.2166	1.14	C21H30O5	[M+H]+	**2**
S-adenosylmethionine (SAMe)	398.13724	399.145	7.21	C15H22N6O5S	[M+H]+	**2**
1-Stearoyl-2-linoleoyl-sn-glycerol	620.53798	621.5453	0.72	C39H72O5	[M+H]+	**1**
Phosphatidylcholine (PC 34:1)	759.57781	760.5815	1.25	C42H83NO8P+	[M+H]+	**1**
Phosphatidylserine (PS)	791.56763	792.5749	1.48	C42H82NO10P	[M+H]+	**1**
Acetyl-CoA	809.12577	809.8101	14.74	C23H38N7O17P3S	[M+H]-	**2**
Kynurenine	208.08479	207.078	4.4	C10H12N2O3	[M-H]	**2**
Leu-Gly-Gly	245.13756	246.1448	1.14	C10H19N3O4	[M+H]+	**2**
