# Supplementary figures and images for: Metabolic Reprogramming in Response to Freund’s Adjuvants: Insights from Serum Metabolomics
Source: Microorganisms. 2025 Feb 22;13(3):492. doi: 10.3390/microorganisms13030492 (PMC11944801; doi:10.3390/microorganisms13030492)

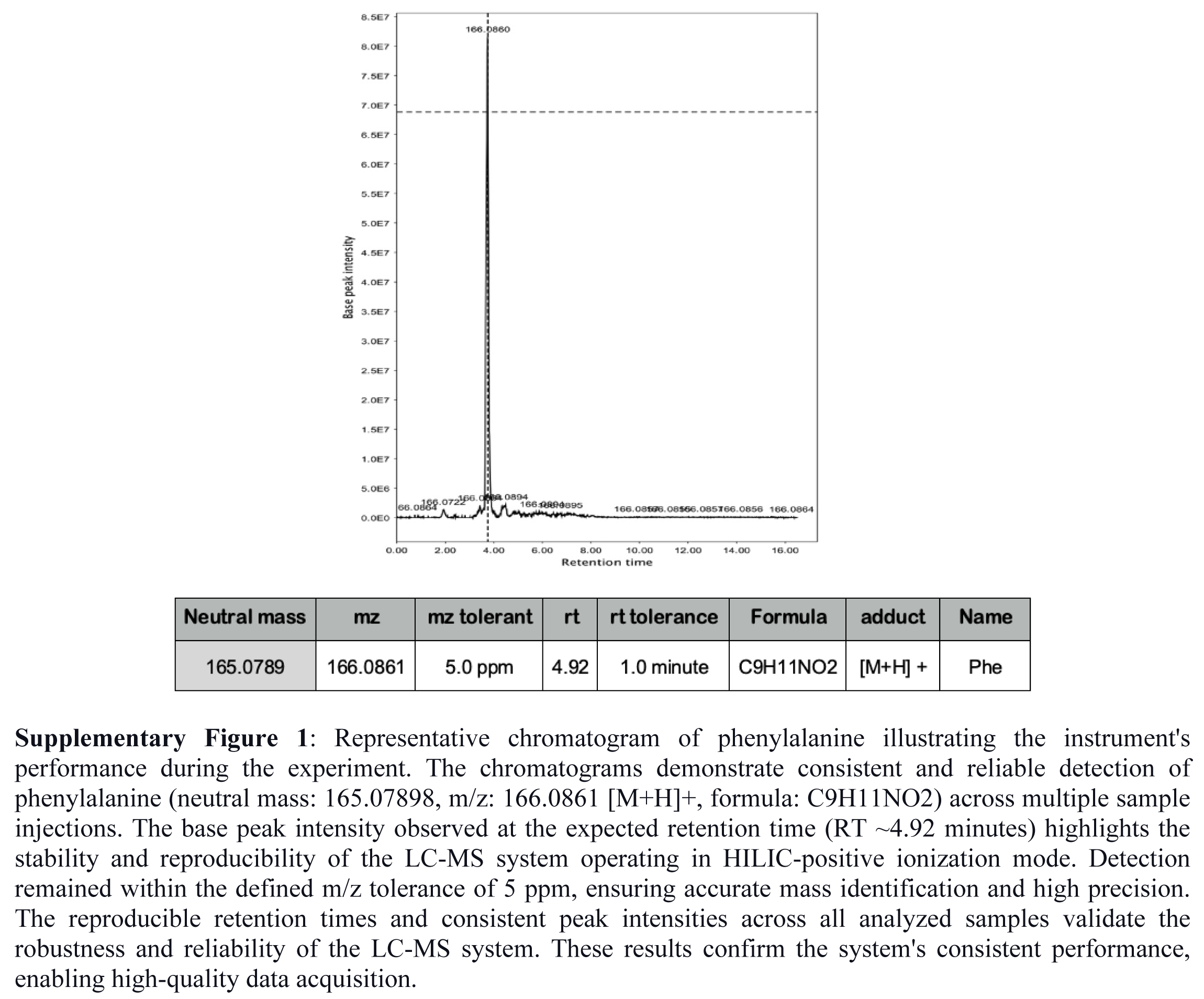

Supplement: Supplementary file 1 [file microorganisms-13-00492-s001.zip › Figure S1.tif]

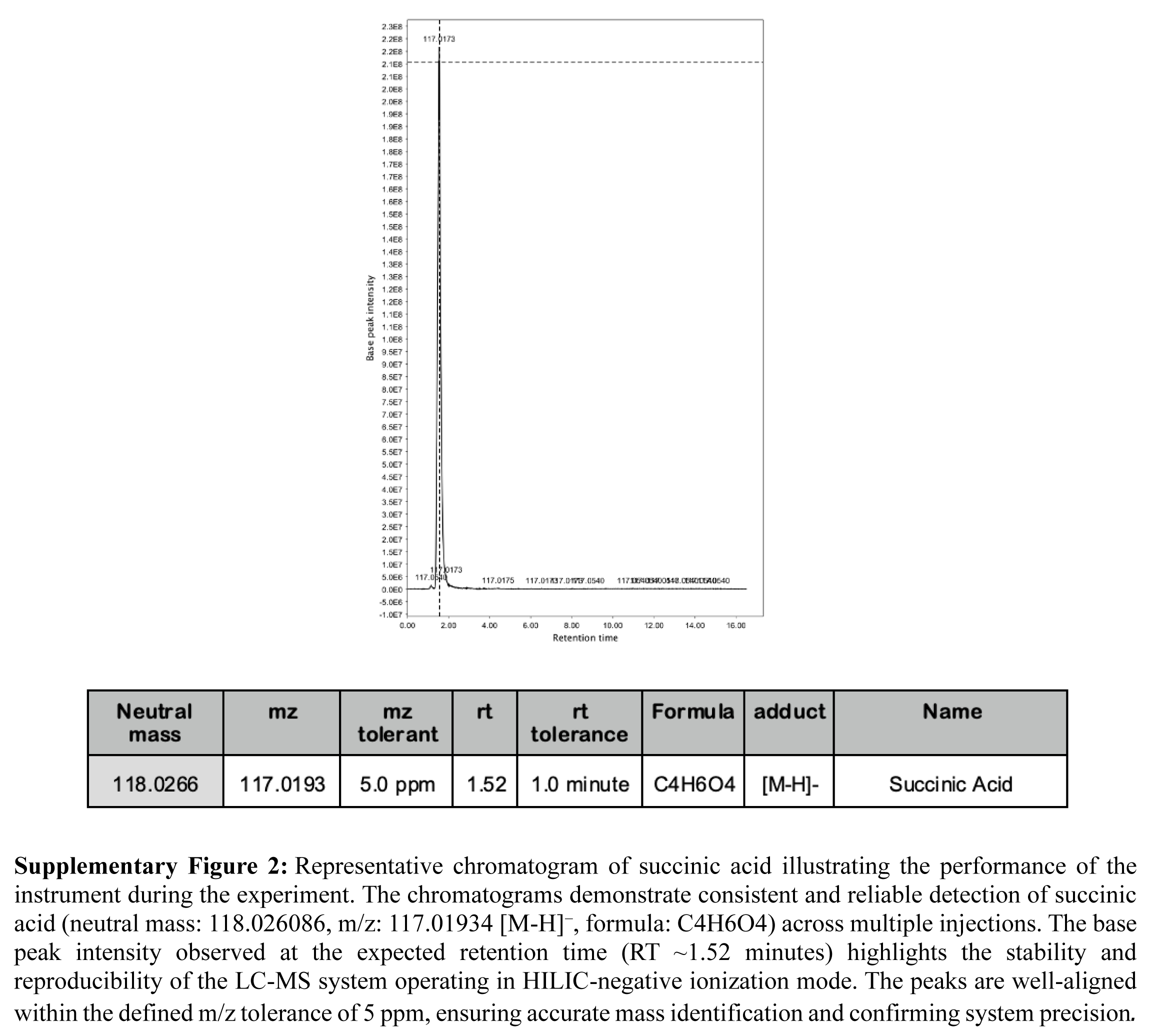

Supplement: Supplementary file 1 [file microorganisms-13-00492-s001.zip › Figure S2.tif]

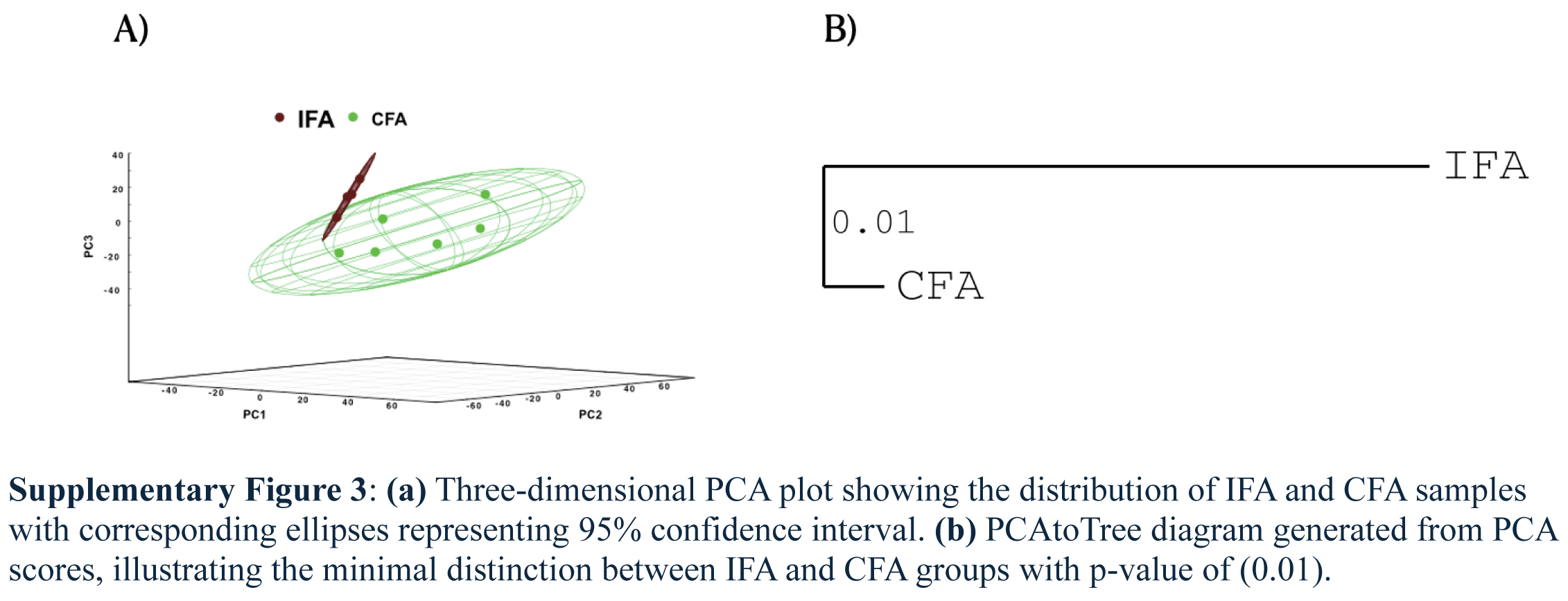

Supplement: Supplementary file 1 [file microorganisms-13-00492-s001.zip › Figure S3.tif]
